# Botanic Garden as a Factory of Molecules: *Myrtus communis* L. subsp. *communis* as a Case Study

**DOI:** 10.3390/plants11060754

**Published:** 2022-03-11

**Authors:** Claudia Giuliani, Martina Bottoni, Fabrizia Milani, Sefora Todero, Patrizia Berera, Filippo Maggi, Laura Santagostini, Gelsomina Fico

**Affiliations:** 1 Department of Pharmaceutical Sciences, University of Milan, Via Mangiagalli 25, 20133 Milan, Italy; claudia.giuliani@unimi.it (C.G.); martina.bottoni@unimi.it (M.B.); fabrizia.milani@unimi.it (F.M.); sefora.todero@studenti.unimi.it (S.T.); 2 Ghirardi Botanic Garden, Department of Pharmaceutical Sciences, University of Milan, Via Religione 25, 25088 Toscolano Maderno, Italy; segreteria@reteortibotanicilombardia.it; 3 Rete degli Orti Botanici della Lombardia, Piazza Matteotti 27, 24100 Bergamo, Italy; 4 School of Pharmaceutical and Health Products Sciences, University of Camerino, Via Madonna delle Carceri 9, 62032 Camerino, Italy; filippo.maggi@unicam.it; 5 Department of Chemistry, University of Milan, Via Golgi 19, 20133 Milan, Italy; laura.santagostini@unimi.it

**Keywords:** myrtle, botanic gardens, secretory cavities, essential oils, α-pinene, 1,8-cineole and linalool chemotype, light microscopy, GC-MS, Open science

## Abstract

A novel perception of botanic gardens as complex “factories of molecules” (Lombardy Region Project–Lr. 25/2016, year 2021), that mediate plant–environment interactions, and are the basis of their utility for humans, is presented. The core-topic is the medicinal plant heritage of the Ghirardi Botanic Garden (Toscolano Maderno, Brescia, Italy) of the University of Milan. In this work, we studied *Myrtus communis* L. subsp. *communis* (Myrtaceae) at multiple scale levels: macro- and micromorphological, with special emphasis on the secretory structures responsible for the production of secondary metabolites; phytochemical, with the analysis of the essential oil (EO) composition from leaves (fresh, dried, stored at −20 °C and at −80 °C) and fruits over two consecutive years (2018 and 2019); bio-ecological, with a focus, based on literature data, on the ecology and biological activity of the main EO components. The occurrence of secretory cavities producing terpenes, along with flavonoids, was proven. A high level of chemical variability across the obtained EO profiles emerged, especially that concerning quantitative data. However, regardless of the different conservation procedures, the examined plant part, or the phenological stage, we detected the presence of three ubiquitous compounds: α-pinene, 1,8-cineole, and linalool. The overall results will serve to enrich the Ghirardi Botanic Garden with novel labeling showing accurate and updated scientific information in an Open science perspective.

## 1. Introduction

Botanic gardens currently achieve numerous missions related to biodiversity conservation, scientific research, and educational and dissemination activities [1]. Specifically, thanks to their competences in research and public engagement, university-based botanical gardens have a great under-exploited potential in fostering activities such as knowledge transfer and community service, contributing to society’s social and cultural development and in creating interactions between academia and the territory [2].

Within this framework, the present work aimed at offering a new perception of botanic gardens as complex “factories of molecules” that mediate the interactions of the plant world with the environment, and that are at the basis of their utility for humans. The core-topic of our study is the plant heritage preserved at the Ghirardi Botanic Garden (Department of Pharmaceutical Sciences, University of Milan, Toscolano Maderno, Brescia, Italy), where only medicinal species from all over the world are preserved, and where a dedicated project is underway (“Ghirardi Botanic Garden, factory of molecules”—Lombardy Region Project, L.r. 25/2016, year 2021).

**Figure 1:**
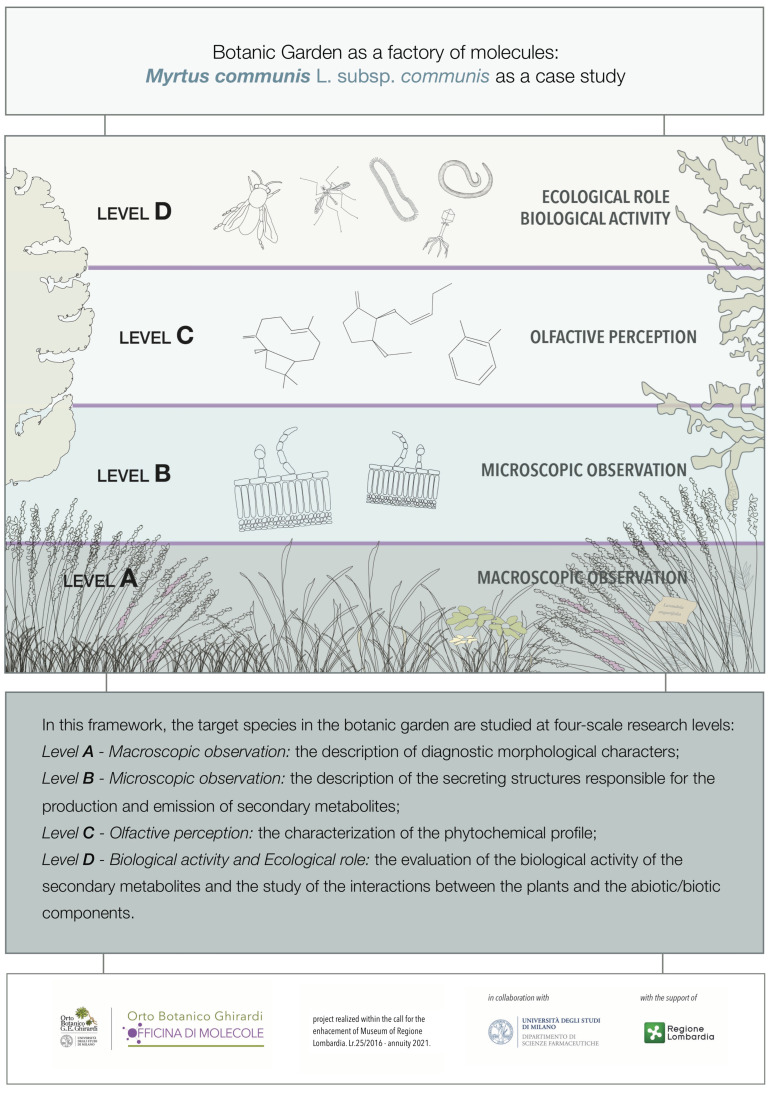
Main iconography of the project “Ghirardi Botanic Garden, factory of molecules”.

In this project, the botanic garden, with special reference to selected target-*taxa*, was studied at four-scale research levels (Figure 1): (a) macroscopic, through the description of diagnostic macromorphological features; (b) microscopic, through the study of the secreting structures responsible for the production and emission of secondary metabolites; (c) phytochemical, through the characterization of their profile; (d) bio-ecological, regarding the evaluation of their biological activity and ecological significance. 

Finally, the research actions were declined in scientific dissemination activities, according to the University Third Mission, with special regard to the public of the botanic garden.

In this paper, we addressed our attention on myrtle (*Myrtus communis* L. subsp. *communis*, Myrtaceae), preserved at the Ghirardi Botanic Garden since its foundation in 1964, thus becoming the *viaticum* to describe the “philosophy” of the project.

*Myrtus communis* subsp. *communis* is an evergreen shrub or a small tree [3], growing spontaneously throughout the Mediterranean basin. The stem is branched from the basal portion and the bark is brownish or reddish in color. The leaves are simple, opposite, sessile or sub-sessile, glossy, and dark green in color, lanceolate or ovoid-elliptical in shape with entire or slightly revolute margins and acute apices; they are very aromatic due to the presence of numerous secretory cavities. The flowers, white in color with yellowish streaks, are solitary or coupled at the leaf axil. The fruits are ellipsoidal or subspherical berries, red-violet or blackish in color at maturity, with persistent calyx residues. 

Myrtle has a consolidated ethnobotanical tradition and is used in different parts of the world, against colds and coughs [4], for self-medication of digestive problems, for skin disorders [5], for the treatment of obesity, hypercholesterolemia, and diabetes [6,7]. The essential oils (EOs), obtained from shoots, leaves and sometimes flowers and berries, are used eminently in perfumery. The berries are also employed in the production of bitters and famous liquors. Due to its broad use in folk medicine, myrtle has been widely investigated, especially with regards to the EO composition [8,9,10,11,12,13,14]. In Italy, previous studies were focused on plants from Sardinia, Sicily, Campania, and Liguria regions [11,12,15,16,17,18]. Concerning literature data on the micromorphology, previous investigations were focused on the structure and ontogeny of the secretory cavities of leaves and flowers by means of both light and electronic microscopy [19,20,21,22].

Literature contributions concerning the biological activity refer to study on the antioxidant and antimicrobial properties, [23,24,25,26,27,28,29,30], along with potential anti-inflammatory and antitumoral activities [24,25]. Mahmoudvand et al. [31] also documented the relevant antiparasitic activity of the leaf EO towards strains of *Leishmania tropica*.

Recent studies revealed that the fruits and seeds are very rich in phospholipids, polyunsaturated fatty acids, and phenolic compounds, responsible for the effectiveness in the treatment of digestive disorders, esophagitis, diarrhea, and ulcerative colitis [32]. Previous contributions regarding the biotic interactions of myrtle mediated by secondary metabolites are lacking.

In this work we investigated *Myrtus communis* subsp. *communis* preserved at the Ghirardi Botanic Garden in the context of a transversal and multidisciplinary project, where the focus of the scientific research plans is driven by the public perspective. We performed: (i) a micromorphological and histochemical investigation on the secretory cavities of leaves and shoots; (ii) the analyses of the EO compositions obtained from different plant parts and following different conservation procedures over two following years, 2018 and 2019; (iii) the correlation of the EO profiles with the potential biological and ecological role through the analysis of literature data. Finally, the results of our study converged in the realization of a novel interpretative apparatus that shows the visitors of the garden updated details of the research, performed in an Open science context, in the attempt to strengthen the link between scientific findings and society.

## 2. Results and Discussion

### 2.1. Micromorphological Investigation

**Figure 2:**
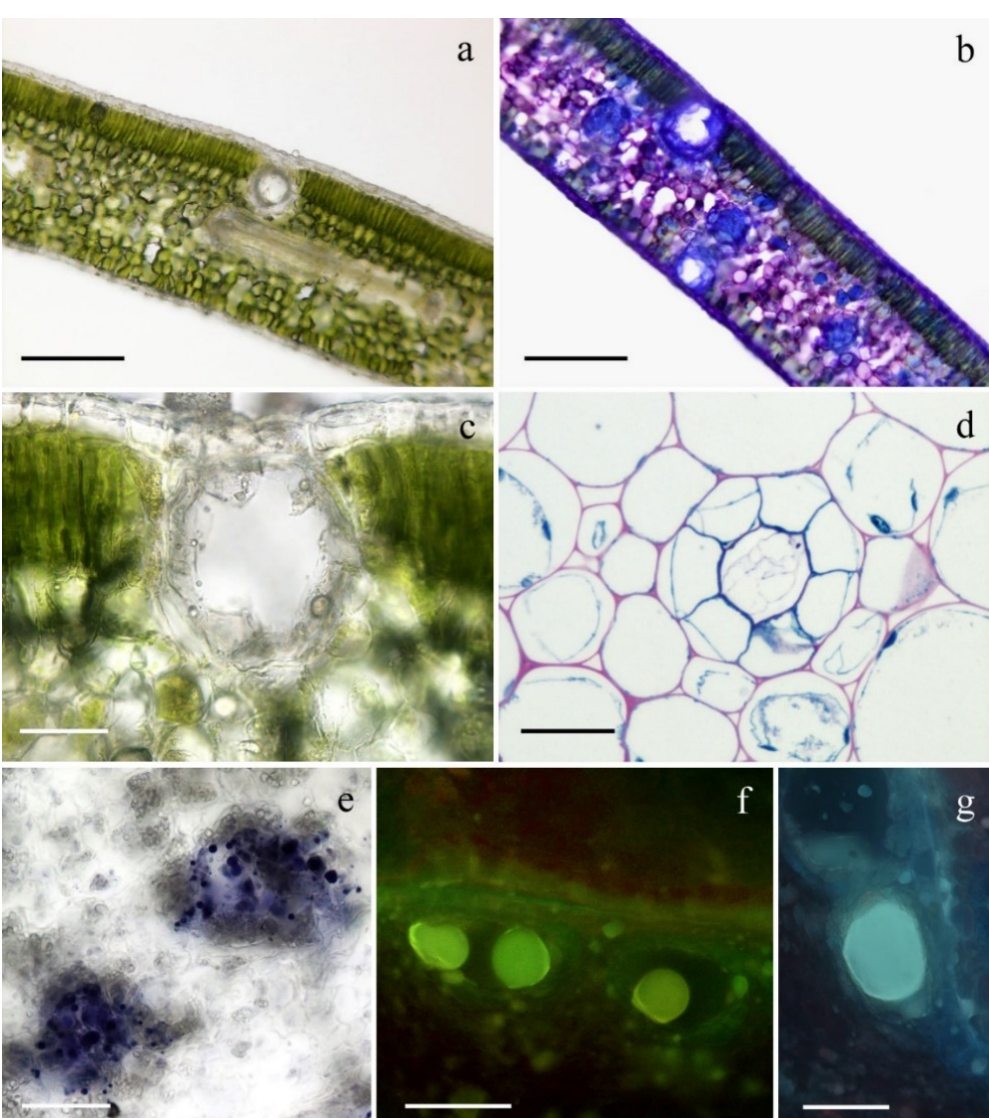
(**a**,**b**) Cross section of *Myrtus communis* subsp. *communis* leaf colorless (**a**) and stained with Toluidine blue dye, (**b**); (**c**) Detail of a leaf secretory cavity, colorless; (**d**) Detail of a shoot secretory cavity; (**e**–**g**) Histochemistry of secretory cavities: Nadi reagent (**e**), Fluoral Yellow-088 (**f**), AlCl_3_ (**g**). Scale bars = 100 µm (**a**,**b**); 25 µm (**c**,**d**,**g**); 50 µm (**e**,**f**).

The micromorphological survey showed the occurrence of secretory cavities both in the leaves and in the shoots. In the leaves these structures were variously distributed: in the palisade parenchyma, where they are located immediately below the adaxial epidermis; in the spongy parenchyma, and especially in the transition region with the palisade mesophyll or adjacent to the abaxial epidermis (Figure 2a,b). In the shoots, the cavities had a reduced diameter and generally occurred in the cortical parenchyma.

The secretory cavities, regardless of the distribution pattern, were globose-spheroidal in shape and had a diameter ranging from 10 to 50 µm (Figure 2c). The cavities displayed a 1-layered epithelium consisting of secreting cells that released the secreted material inside the cavity (Figure 2d). The cavities generally appeared empty, but sometimes the accumulation of colorless or pale-yellow material was observed; the secreted material consisted of small, densely packed droplets or of large clusters which filled the entire volume of the cavity. These results are in accordance with those reported in the literature [19,22]. 

The histochemical tests revealed consistent results between the cavities of leaves and shoots (Figure 2e–g). All the dyes specific for lipophilic substances gave positive responses, with special reference to the NADI reagent indicating the presence of terpenes (Figure 2e,f). Furthermore, the production of flavonoids was for the first time highlighted in the cavities of *M. communis* subsp. *communis* following the treatment with AlCl_3_ (Figure 2g), while the tests specific for polysaccharides and proteins gave negative results.

### 2.2. Phytochemical Investigation

The EO compositions of *M. communis* subsp. *communis* were assessed over two consecutive years (2018 and 2019), according to different objectives.

*(i)*  In 2018, we analyzed the leaves to evaluate the EO compositions, following different preservation methods (Table 1).
*(ii)*  In 2019 we analyzed air-dried samples according to the following considerations: the highest EO yield; the easiest storage procedure; the evidence that the literature contributions indicated air-drying as the most usual conservation method. Taking into account these points and the awareness of the variability in the EO profile compared to the fresh material, we investigated the EO compositions from air-dried leaves collected at two different stages of the plant cycle, vegetative and reproductive (Table 2). We further characterized the profile of the fruits at two collection times: early fruiting and ripening (Table 3).

In 2018 the leaves were treated according to four different preparation methods and separately hydro-distilled: as fresh material (*FL*), after freezing at −20 °C (*20FL*), after freezing at −80 °C (*80FL*), and after air-drying at room temperature (*DL*). The profiles of the leaf EOs are reported in Table 1. 

The obtained oil yields ranged from 0.36% in the fresh samples up to 1.08% in the dried ones. The samples stored at −20 °C and at −80 °C showed similar values (0.49% and 0.46%).

The more complex profile, due to the presence of the highest number of total compounds, was that obtained from *FL* (46), followed by *80FL* (38). The other two leaf samples displayed 26 (*20FL*) and 25 (*DL*) total compounds. However, the additional compounds in *FL* occurred in amounts ranging from 0.1% up to 0.9%. 

The dominant compound classes were monoterpenes, accounting for almost the total relative abundances (*FL* 92.9%, *80FL* 97.5%, *20FL* 96.4%, *DL* 95.9%). The other classes exhibited total relative abundances lower than 7.0% in *FL* and lower than 4.5% in all the other samples. The principal compounds were invariably α-pinene (*5*, *FL* 38.6%, *80FL* 36.2%, *20FL* 41.2%, *DL* 41.6%), followed by 1,8-cineole (*15*, *FL* 31.0%, *80FL* 25.5%, *20FL* 28.2%; *DL* 26.1%), linalool (*20*, *FL* 10.7%, *80FL* 28.7%, *20FL* 18.7%, *DL* 18.2%) and α-terpineol (*28*, *FL* 1.8%, *80FL* 2.1%, *20FL* 1.4%, *DL* 2.0%).

The number of compounds common to all the examined leaf samples was established as 22, including the dominant ones. Considering the two more complex profiles, *FL* and *80FL*, 14 common compounds were detected, all occurring with amounts lower than 1.0%, except for *trans*-geranil (*30*, 1.3%) in the fresh leaves. The *FL* profile presented seven exclusive compounds, i.e., *cis*-sabinene hydrate (*14*), nerol oxide (*23*), pinocarvone (*24*), aromadendrene (*38*), 2-tridecanone (*40*), *trans*-nerolidol (*42*), and 5,8,11-heptadecatrien-1-ol (*46*), all accounting for amounts lower than 0.7%. The other profiles were not characterized by the occurrence of exclusive compounds; however, the presence of limonene (*13*) in *20FL* (2.9%) and in *DL* (3.3%) was noteworthy. The freezing of the material at −80 °C would appear to preserve the EO qualitative composition of the fresh plant matrix. The consistent decline of the total compounds after freezing at −20 °C and air-drying suggests the degradation of some of them and the transformation of others. 

Referring to the drying procedure, probably due to degradation processes, a relationship has been documented between limonene and oxygenated monoterpenes, with a decrease in the relative percentages of geraniol and terpineol derivatives and a simultaneous increase in the concentration of limonene [33]; observing our data for *FL* and *80FL* (Table 2), the sum of those derivatives (*25*, *27*, *32*, *33*, *35*) should be compared to the increasing abundance of limonene (*13*). 

Based on the results of the first phase of our work, it emerges that although the minor molecules rarely reached a relative percentage of 1.0%, the profiles of the dried material (usually used and described as the starting plant matrix in literature) were lacking in the quantity of compounds.

The variability in EO profiles among the different plant matrices, aroused our curiosity and therefore a targeted literature survey on ecological topics was conducted. With regards to the main common compounds, α-pinene (*5*) is mostly associated with repulsion mechanisms against numerous herbivorous insects, very often in synergy with 1,8-cineole or linalool [34]. However, it is also an attractant for common large wood-boring beetles from southeastern USA, being used in traps to detect and monitor these pests in forested areas [35]. 1,8-Cineole (*15*) shows anti-bacterial activity [36] and a powerful toxicity against important wheat pests [37]; however, it is also recognized as an attractant of different bee pollinators [38,39]. Linalool (*20*) is documented to intoxicate and repel herbivores [40] and possess antibacterial and antifungal activity [41]: however, it also attracts the males of *Ceratitis capitata*, one of the most economically important fruit fly pests [42]. α-Terpineol (*28*) exhibits insecticidal properties [43] and is emitted from the bark of *Pinus sylvestris* L. to deter the black pine sawyer beetle, a serious pest [44]. However, literature refers also to α-terpineol as a pest attractant of *Megacyllene antennata*, a species native to southwestern North America whose larvae feed on woody tissues of mesquite [45]. 

Concerning the exclusive compounds of the fresh leaves, deterrent actions were documented for *cis*-sabinene hydrate (antimicrobial, [46]), aromadendrene, and pinocarvone (larvicidal, [47]); *trans*-nerolidol represents a powerful signal that stimulates the expression of plant defense towards herbivores and different types of parasites [48]. For the remaining compounds literature data were lacking. 

Therefore, the defensive role of the compounds present in the leaf EOs seems to emerge.

In 2019, the *DL* EOs referring to two different stages of the plant cycle displayed a high level of consistency (Table 2). The oil yields were similar (0.96%, 1.02%). Both were characterized by 38 different total compounds; unexpectedly, the profile was richer than in 2018 with an increase from 25 to 38 total compounds—this underlines the qualitative variability in the profiles in different years in relation to the presence of some minor compounds (with relative percentages lower than 1.0%). The oxygenated monoterpenes (67.4–68.8%) dominated on monoterpene hydrocarbons (23.0–28.9%), and the other compound classes accounted for a total percentage of about 5.0% in March and of about 7.5% in October. The principal compounds were the same even if with different relative percentages: 1,8-cineole (*12*, 23.6%, and 35.1%), linalool (17, 35.8%, and 25.2%), α-pinene (*4*, 27.4% and 19.5%), and α-terpineol (*20*, 3.0%, and 2.0%). Only 1,8-cineole increased considerably, whereas the other main compounds declined from the beginning of the vegetative stage (March) up to fruit ripening (October). Their prevailing deterrent roles fit entirely with the need to protect the young leaves in a susceptible developmental phase, i.e., the onset of the vegetative phase in March. However, considering the increased trend of 1,8-cineole in the two investigated phenological phases and its high relative percentage at fruit ripening, it could be assumed that at blooming this compound could be also connected to the enhancement of the attraction towards pollinators, operated by flowers.

Differently from 2018 *DL*, 2019 *DL* did not produce limonene (*13*). As regards to the ecological role of this compound, it serves as an antifeedant, an antifungal and acts as an oviposition deterrent for many herbivores [49,50]. As an example, in a study on *Pinus sylvestris* L., authors documented that in both resistant and susceptible *cultivars* to the pine moth herbivore, *Dioryctria zimmermani*, the production of monoterpenes varies considerably when the plants are attacked: limonene was the only compound that was consistently higher in resistant *cultivars* [51]. 

As regards to fruits (Table 3), we compared samples collected in July (early fruiting stage) and October (ripening stage). The oil yields were similar, being 0.59% and 0.48%, respectively. The fruit profiles showed 56 total compounds in July and 51 in October; the additional compounds of July EO occurred in amounts lower than 0.4% or in traces. The monoterpenes dominated (77.2%, 79.5%), followed by appreciable amounts of sesquiterpenes (9.2%, 14.9%). The most abundant compounds (taking into account relative percentages higher than 5.0%) are the following: α-pinene (*5*, 11.9%), 3-carene (*10*, 6.5%), *o*-cymene (*12*, 7.6%), 1,8-cineole (*14*, 6.4%), γ-terpinene (*16*, 5.1%), α-terpinolene (*18*, 5.2%), linalool (*20*, 8.8%), and α-terpineol (*28*, 6.1%) in July; α-pinene (*5*, 21.4%), *o*-cymene (*12*, 7.9%), limonene (*13*, 6.8%), 1,8-cineole (*14*, 12.2%), and linalool (*20*, 9.4%) in October.

In the transition from the early fruiting to the ripening stages, we detected a wide quantitative variability for the main compounds, according to the following patterns: α-pinene (*5*), limonene (*13*), 1,8-cineole (*14*) increased considerably, and linalool (*20*) underwent a more limited rise; 3-carene (*10*), α-terpinene (*11*), γ-terpinene (*16*), α-terpinolene (*18*), and α-terpineol (*28*) declined; the amounts of *o*-cymene (*12*) were similar. 

In the shift from the unripe to the ripe stages, only quantitative differences emerged, with increased production of such compounds that were present in low proportions in unripe fruits or vice versa, and with declined production of others. The involvement in defensive mechanisms was mainly documented for the molecules of the fruit profiles which underwent quantitative fluctuations from the unripe to ripe stages, regardless of the trends of decline or increase. These fluctuations can be explained not only in the defense towards microorganisms, phytophagous insects, or herbivores, but also in tri-trophic interactions. Therefore, we could hypothesize that attraction strategies towards insects capable of defending the plant from parasites are activated. For example, we cite the following compounds: 3-carene (*10*, herbivore-induced attractant of *Nesidiocoris tenuis*, a predator of major tomato pests [52], *o*-cymene and γ-terpinene (*12*, *16*, antimicrobials, [53,54]), α-terpinolene (*18*, antifeedant, [55,56]); limonene (*13*, attractant for pest and pathogen attraction during orange ripening, facilitating access to the fruit for pulp consumers and seed dispersers [57].

The fruits showed 14 exclusive compounds, all present with percentages lower than 0.3% except for *E*-β-ocimene (*15*, 4.3%, 1,3%). 13 of these compounds occurred in both collection times, whereas ocimenol (*27*) and γ-elemene (*40*) were only present at the unripe stage.

*E*-β-ocimene (*15*) is known to play a crucial role in attracting insect pollinator in floral blends [58], but it is also a herbivore-induced monoterpenoid acting by giving airborne signals to nearby plants in response to insect attack [59]. Moreover, it is a common aphid alarm pheromone that is released by attacked aphids and causes other aphids in the vicinity to stop feeding and move away [60].

With regard to the major complexity of fruit profiles in comparison to leaves, this pattern is exactly that expected where the fruit organoleptic properties are used by animals to find or identify ripe fruits [61], as plants are expected to be selected to begin attracting them only after the seeds are viable. However, myrtle seeds are mostly dispersed by frugivorous birds, i.e., passerines [62,63] that rely mainly on vision for the detection and selection of ripe fruits [64]. 

Moreover, comparing leaf and fruit profiles and considering the evolution of the three major compounds, we note that only 1,8-cineole increased, showing the same pattern in both plant parts. We wonder if the increase of this compound even in ripe fruits can attract seed dispersers or has defensive action against pathogens by adding its activity to that of the other main compounds. In addition, several minor compounds also underwent fluctuations across the ripening period. We cannot exclude the minor compounds from these assessments, since their relevance would result in the expression of synergistic mechanisms underlying the repulsive roles of the most abundant compounds.

At the same time, in multiple trophic chains for insect herbivores and pathogens and seed-dispersing vertebrates, even compounds which are defensive in other tissues or play a defensive role in fruits may primarily be selected due to their secondary function in attracting seed dispersers. For example, limonene, which increased by 8-times at fruit ripeness, dominates the scent of ripe oranges and was considered to play a defensive role, but is in fact an attractant of vertebrate and invertebrate antagonists [57], as stated before. 

With regards to the literature contributions on myrtle EO composition, in Table S1 we depicted the main compounds (relative percentages higher than 9.5%), detected in the samples from different European and extra-European countries [8,9,11,12,15,16,17,18,23,30,65,66,67,68,69,70,71,72,73,74,75,76,77,78,79,80,81,82,83,84,85,86,87,88,89,90,91,92,93,94,95,96,97,98,99,100,101,102,103,104,105,106,107,108,109,110,111,112,113,114,115,116,117,118,119,120,121,122,123,124,125,126,127,128,129,130,131,132,133,134,135,136,137,138]. The most investigated samples were those from Iran and Tunisia. Leaves resulted as the most studied plant material, though fruits and aerial parts were also the target of study by many researchers. Shoots and flowers, not investigated herein, were the subject of each of three previous contributions. The drying process resulted as the most common conservation procedure.

With regard to the chemical composition, myrtle EOs were predominantly constituted by: (a) α-pinene and 1,8-cineole; (b) α-pinene, 1,8-cineole and linalool; (c) α-pinene, 1,8-cineole, limonene and linalool; (d) myrtenyl acetate; (e) 1,8-cineole, linalool, and myrtenyl acetate; (f) 1,8-cineole, α-pinene, and myrtenyl acetate; (g) 1,8-cineole, limonene and myrtenyl acetate; (h) α-pinene, 1,8-cineole, limonene, linalool, and myrtenyl acetate. The presence of α-terpineol is also frequent in myrtle EOs in the literature, as was found in our samples. Other components occurred also in high amounts in myrtle EOs with different origins, although only in very few cases (e.g., linalyl acetate, methyleugenol, β-caryophyllene, camphene, (*E*)-β-ocimene, myrtenol) (Table S1). The first three components also occurred in all our samples in relative amounts around 1.0%, except for linalyl acetate and β-caryophyllene that doubled and halved, respectively in the EO profile of ripe fruits with respect to the unripe stage; camphene was present in percentage lower than 0.5% and the last two compounds were lacking. 

According to literature, the factors that can influence the chemical composition of myrtle EOs included the following: geographical origin, plant material used, conservation method, analytical method, plant phenological stage, wild-growing or cultivated plants, and the existence of different genotypes or chemotypes [65]. 

As a whole, the profiles investigated herein showed the most complex compositions compared to the EOs known from the literature, regardless of the studied plant part. Indeed, our EO profiles showed invariably the greatest number of several minor compounds. 

Concerning the comparison with the previously investigated Italian samples, our study represents the first survey performed on *M. communis* in Northern Italy. Qualitative and quantitative differences emerged; however, the main compounds were ubiquitous even if present in different relative abundances [11,12,15,16,17,18]. Our samples were characterized by the highest amounts of α-pinene, which varied in relative percentages according to the conservation method, declining with the drying process. 

In the samples investigated herein, the major components of the EOs obtained from leaves and berries were invariably α-pinene, 1,8-cineole, and linalool. Noteworthy was the exclusive presence of limonene in the air-dried and −20 °C stored leaves of 2018 and in the ripe fruits. Therefore, the target-species cultivated at the Ghirardi Botanic Garden belongs to the α-pinene, 1,8-cineole, and linalool chemotype and is characterized by the lack of myrtenal acetate. The other investigated Italian samples, from Sardinia, Campania, Liguria, and Sicily belonged instead to the α-pinene, 1,8-cineole, and limonene and linalool chemotype [11,12,15,16,17,18]; however, it was ascertained that all the Italian samples lacked myrtenal acetate. This ester was found on the contrary in the myrtle EO from various Mediterranean countries, i.e., Tunisia, Morocco, Albania, Croatia, Montenegro, and Turkey (Table S1). 

Finally, a literature survey was also performed on the biological activity ascribed to myrtle EOs. Different authors referred to antioxidant [23,24], antifungal [26,27,28]), and antimicrobial properties [65]. Anti-mutagenic effects [27] and anticancer activity of the EOs against P815 and MCF-7 tumor cell lines [24] were also documented, along with anti-diabetic properties and the effect on LDL oxidation [24,25]. Previous in vitro studies also documented the relevant antiparasitic activity of the leaf EO of myrtle towards strains of *Leishmania tropica* [31]. 

### 2.3. Scientific Dissemination

**Figure 3:**
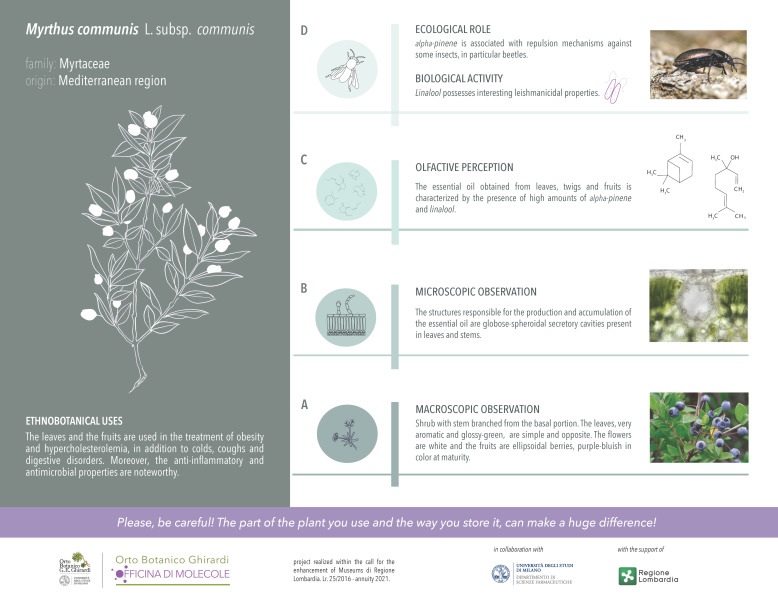
New labelling of *Myrtus communis* L. subsp. *communis* at the Ghirardi Botanic Garden (University of Milan, Toscolano Maderno, Brescia, Italy).

The scientific results reported in the “Micromorphological investigation” and “Phytochemical investigation” sections converged in the realization of a new interpretative and iconographic apparatus for *M. communis* L. subsp. *communis* at the Ghirardi Botanic Garden (Toscolano Maderno, Brescia, Italy). In addition to the plant macroscopic features, it highlights the microscopic morphology, the main components of the EO profile, along with data concerning their ecological roles and biological activity (Figure 3).

As a future work perspective, we will add a specific QR Code, a machine-readable barcode label, referring to information stored by URLs, i.e., plain text, images, geolocation.

## 3. Materials and Methods

### 3.1. Plant Material

*Myrtus communis* L. subsp. *communis* is cultivated at the Ghirardi Botanic Garden, Department of Pharmaceutical Sciences, University of Milan (Toscolano Maderno, Brescia, Italy). Prof. G. Fico identified the plant according to Pignatti et al. [3].

The samplings were performed over two consecutive years, 2018 and 2019 (Table 4). In 2018, leaves were collected and divided into four aliquots, each subjected to a different storage procedure, i.e., hydro-distilled as fresh material, freezing at −20 °C, freezing at −80 °C and drying at room temperature away from direct sunlight for 30 days. In 2019, leaves were gathered at two different times, March, and October, corresponding to different stages of the plant cycle, vegetative and reproductive; then they were air-dried. Moreover, fruits were gathered and dried at two different collection times, early fruiting (July) and ripening (October).

Voucher specimens were labelled with the codes GBG2018/048 and GBG2019/052 and deposited in the Herbarium of the Ghirardi Botanic Garden.

### 3.2. Chemicals

Solvents were of gradient grade purity and purchased from either Exacta Optech Labcenter SpA (San Prospero (MO), Italy) or VWR International (Milan, Italy). All the reagents were of reagent grade purity, purchased from Sigma Aldrich (Merck group, Milan, Italy), Fisher Scientific Italy (Rodano (MI), Italy), or VWR International (Milan, Italy), and used as received.

Observations by Light and Fluorescence microscopy were performed under a Leitz DM-RB Fluo Optical Microscope equipped with a Nikon DS-L1 digital camera.

### 3.3. Micromorphological Investigation

#### Light Microscopy and Fluorescence Microscopy

The micromorphological investigation under Light microscopy and Fluorescence microcopy was performed on leaves and shoots, collected from the same individual, in July 2018. We used both fresh material and fixed samples included in historesin (Technovit^®^ 7100). 

For the fresh material, sections of thickness ranging from 30 to 50 µm were obtained for fresh leaves using a vibratome, and for shoots using a cryostat.

Samples were also fixed in F.A.A. solution (formaldehyde:acetic acid:ethanol 70% = 5:5:90) for 10 days at 4 °C. Subsequently, fixed samples were washed in 70% ethanol for 24 h; they were then dehydrated progressively by treatment with 80% ethanol for 2 h, 95% ethanol for 2 h, and then twice in absolute ethanol for 2 h/each. Pre-inclusion was then performed first with ethanol and historesin in a 1:1 ratio for one night, then with a 1:2 ratio for 2 h and in pure historesin for 3 h. Finally, the inclusion was done in a polypropylene capsule with addition of hardener in a ratio 1:15 of basic resin. The historesin samples were cut in 2 µm-sections, by an ultramicrotome.

At least 10 replicates for leaves and shoots were collected in each year of investigation to evaluate the level of variability in the morphology, distribution, and histochemistry of the secretory cavities. The following dyes were used: Fluoral Yellow-88 for total lipids; Nile Red for neutral lipids; Nadi reagent for terpenes; Alcian Blue for mucopolysaccharides; Ruthenium Red for pectins; ferric trichloride for polyphenols; aluminum trichloride and Naturstoff-reagenz-A for flavonoids.

### 3.4. Phytochemical Investigation

#### 3.4.1. Preparation of Essential Oils (EOs)

For each harvest period, the dried, frozen, or fresh plant material was weighed, roughly chopped, and ground, and then subjected to hydrodistillation in a Clevenger apparatus.

The fresh, frozen, and air-dried samples of myrtle of the years 2018 and 2019 were ground, put into 4 L or 2 L flasks filled with deionized water, with a 1:10 plant material/water ratio, and subjected to hydrodistillation using a Clevenger-type apparatus for 2 h, checking that after this time the volume of oil obtained remained constant. Once obtained, the essential oil was decanted and separated from water, and residual drops were removed using anhydrous sodium sulphate. The oil yield was estimated on a fresh weight basis (*w/w*) for fresh and frozen samples and on a dry weight basis (*w/w*) for the dried ones. Due to the complex investigation approach proposed and the presence of only one specimen of myrtle in the botanic garden, no replicas were performed for distillation. Replicas were performed in sampling of fresh leaves, to evaluate variation in essential oil composition; results showed only small variations in relative amounts of single components, which did not affect the percentage data reported in Table 1. 

#### 3.4.2. GC-MS Analysis of Essential Oils

Essential oils were analyzed by GC-MS using two diverse instruments. The first instrument used was an Agilent 6890 N equipped with a 5973 N mass spectrometer. Separation was achieved on an HP-5 MS capillary column (5% phenyl-methyl-polysiloxane, 30 m, 0.25 mm i.d., 0.1 μm film thickness; J and W Scientific, Folsom, CA, USA) using helium as the carrier gas (1 mL min^−1^). The temperature of the oven was set at 60 °C for 5 min, then raised at 4 °C min^−1^ up to 220 °C, and finally 11 °C min^−1^ up to 280 °C. The TICs were acquired at 70 eV scanning in the 29–400 m z^−1^ range. The oil samples were diluted 1:100 in *n*-hexane, and the volume injected was 2 μL (three injection replicates). Data were analyzed using MSD ChemStation software (Agilent, Version G1701DA D.01.00) and the NIST Mass Spectral Search Program for the NIST/EPA/NIH Mass Spectral Library v. 2.0. The identification of essential oil components was performed by comparison of retention indices, calculated using a C8–C30 series of n-alkanes (Sigma-Aldrich, Milan, Italy) and mass spectra of unknown peaks with those contained in the commercial libraries WILEY275, NIST 08, ADAMS, and FFNSC2 as well as those in a homemade library. Percentage values of essential oil components were obtained from the peak areas in the chromatogram without the use of correction factors.

The second GC-MS instrument was a Thermo Scientific TRACE ISQ QD Single Quadrupole GC-MS. EO separation was performed by a capillary column VF-5ms (5% phenyl-methyl-polysiloxane, length 30 m, 0,25 mm i.d., 0.1 μm film thickness); the temperature gradient was: 8 min at 50 °C, then 4 °C/min until 60 °C, then 6 °C/min from 60 °C to 160 °C and finally 20 °C/min from 160 °C to 280 °C. Injector and detector temperatures were set to 280 °C; carrier gas He, flux 1 mL/min: the mass range detected was 50–500 *m/z*. EO were analyzed pure or diluted 1:100 with n-hexane, with injection volume of 1 µL.

Mass spectra were analyzed by Wiley Mass spectra Library, NIST Mass Spectral Search Program e NIST Tandem Mass Spectral library 2.3; compounds were identified by mass fragmentation and retention index, compared with data stored in mass databases (WILEY, NIST18).

## 4. Conclusions

This multidisciplinary work on *M. communis* subsp. *communis* enabled the following:

*(i)*  to describe for the first time, by digital light microscopy, the distribution pattern of the secretory cavities in full-expanded leaves and shoots—the latter has never been investigated before—and the histochemistry of their secretory products (mainly terpenes, and flavonoids).
*(ii)*  to characterize the profile of EOs obtained across two consecutive years (2018 and 2019) from different plant matrices (leaves and fruits), subjected to different treatments (fresh, −20 °C stored, −80 °C stored, and dried leaves). For leaves, the optimal conservation techniques in relation to the highest oil yield and to the more complex bouquet resulted in air-drying at room temperature and hydrodistillation of fresh and −80 °C/frozen materials, respectively.
*(iii)*  to assign plant growing at the study area to the α-pinene, 1,8-cineole, and linalool chemotypes.
*(iv)*  to speculate, based on literature data, that the main substances produced by leaves and fruits act synergistically for simultaneous protection against pests and pathogens and in attracting natural predators and parasitoids of damaging herbivores, thus protecting plants from further damage.
*(v)*  to channel the scientific results in a novel and original pictorial apparatus for the target *taxon* at the Ghirardi Botanic Garden.

## Figures and Tables

**Table 1:** GC-MS profiles of the essential oils obtained from the leaves of *Myrtus communis* subsp. *communis* collected in July 2018 following different preservation procedures: hydro-distillation as fresh material (*FL*), freezing at −20 °C (*20FL*), freezing at −80 °C (*80FL*), drying at room temperature (*DL*).

	LRI	Class	Compound	Relative Abundance (%)			
				*FL*	*80FL*	*20FL*	*DL*
*1*	769	OTHER	hexanal	0.1	tr	0.1	tr
*2*	844	OTHER	2-hexenal	0.6	0.3	1.2	0.6
*3*	912	OTHER	isobutyric acid, isobutyl ester	1.0	0.4	0.9	1.1
*4*	927	MH	α-thujene	0.1	0.2	0.4	0.6
*5*	935	MH	α-pinene	38.6	36.2	41.2	41.6
*6*	961	MH	camphene	0.2	0.1	0.1	0.1
*7*	983	MH	β-pinene	0.3	0.1	0.2	0.3
*8*	990	MH	myrcene	0.6	0.2	0.3	0.3
*9*	1005	MH	α-phellandrene	0.2	0.2	0.1	tr
*10*	1008	MH	3-carene	0.8	0.2	0.3	0.5
*11*	1019	MH	α-terpinene	0.1	0.1	0.1	0.1
*12*	1028	AH	*o*-cymene	0.1	-	0.3	0.5
*13*	1030	MH	limonene	-	-	2.9	3.3
*14*	1036	MH	*cis*-sabinene hydrate	0.7	-	-	-
*15*	1049	OM	1,8-cineole	31.0	25.5	28.2	26.1
*16*	1052	MH	α-ocimene	0.4	0.3	0.4	0.3
*17*	1064	MH	γ-terpinene	0.5	0.3	0.5	0.4
*18*	1074	OM	*cis*-linalool oxide	0.1	tr	0.2	0.3
*19*	1088	MH	α-terpinolene	0.5	0.5	0.7	0.7
*20*	1100	OM	linalool	10.7	28.6	18.7	18.2
*21*	1125	OM	fenchol	0.1	tr	tr	-
*22*	1140	OM	pinocarveol	0.2	tr	0.2	-
*23*	1143	OM	nerol oxide	0.1	-	-	-
*24*	1154	OM	pinocarvone	0.1	-	-	-
*25*	1159	OM	δ-terpineol	0.1	tr	-	-
*26*	1162	OM	borneol	0.1	tr	-	-
*27*	1168	OM	terpinen-4-ol	0.5	0.2	0.2	0.3
*28*	1202	OM	α-terpineol	1.8	2.1	1.4	2.0
*29*	1248	OM	linalyl acetate	0.9	0.6	0.4	0.8
*30*	1255	OM	*trans*-geraniol	1.3	0.2	-	-
*31*	1296	OM	*trans*-pinocarvyl acetate	0.1	tr	-	-
*32*	1318	OM	methyl geranate	0.1	tr	-	-
*33*	1347	OM	α-terpinyl acetate	0.7	0.3	-	-
*34*	1356	OM	nerol acetate	0.5	0.2	-	-
*35*	1376	OM	geranyl acetate	0.8	0.8	-	-
*36*	1402	OM	methyleugenol	0.9	0.3	-	-
*37*	1423	SH	β-caryophyllene	1.0	0.4	0.5	0.6
*38*	1440	SH	aromadendrene	0.1	-	-	-
*39*	1461	SH	humulene	1.0	0.5	0.4	0.6
*40*	1492	OTHER	2-tridecanone	0.1	-	-	-
*41*	1518	OTHER	durohydroquinone	0.8	0.7	-	0.7
*42*	1562	OS	*trans*-nerolidol	0.4	-	-	-
*43*	1588	OS	caryophyllene oxide	0.4	tr	-	-
*44*	1603	OS	*trans*-bisabolene oxide	0.1	tr	-	-
*45*	1616	OS	humulene oxide II	0.3	tr	-	-
*46*	1660	OTHER	5,8,11-heptadecatrien-1-ol	0.1	-	-	-
*47*	1705	OTHER	methyl ketostearate	tr	0.1	-	-
			*Oil yields*	*0.36%*	*0.46%*	*0.49%*	*1.08%*
			**Total identified**	**98.9**	**99.9**	**99.7**	**100.0**
			**Monoterpene hydrocarbons (MH)**	**42.9**	**38.5**	**47.2**	**48.2**
			**Oxygenated monoterpenes (OM)**	**50.0**	**59.0**	**49.2**	**47.7**
			**Sesquiterpene hydrocarbons (SH)**	**2.1**	**0.9**	**0.9**	**1.3**
			**Oxygenated sesquiterpenes (OS)**	**1.1**	**0.1**	**tr**	**tr**
			**Aromatic hydrocarbons (AH)**	**0.1**	**tr**	**0.3**	**0.5**
			**Other compounds (OTHER)**	**2.7**	**1.5**	**2.1**	**2.4**

The main common compounds are highlighted in grey color. LRI = Linear Retention Index, experimentally obtained on a HP-5MS column using a C_7_–C_30_ mixture of *n*-alkanes.

**Table 2:** GC-MS profiles of the essential oils obtained from the dried leaves (*DL*) of *Myrtus communis* subsp. *communis* collected in March and October 2019.

	LRI	Class	Compounds	Relative Abundance (%)	
				*DL March*	*DL October*
*1*	845	OTHER	2-hexenal	0.1	0.1
*2*	909	OTHER	isobutyric acid, isobutyl ester	0.2	1.0
*3*	925	MH	α-thujene	0.1	tr
*4*	935	MH	α-pinene	27.4	19.5
*5*	952	MH	camphene	tr	0.1
*6*	979	MH	β-pinene	0.1	0.4
*7*	986	MH	myrcene	0.1	0.6
*8*	1004	MH	α-phellandrene	0.1	0.2
*9*	1005	MH	3-carene	0.2	0.8
*10*	1016	MH	α-terpinene	tr	0.1
*11*	1025	AH	*o*-cymene	0.3	0.1
*12*	1034	OM	1,8-cineole	23.6	35.1
*13*	1046	MH	α-ocimene	0.2	tr
*14*	1060	MH	γ-terpinene	0.3	0.7
*15*	1074	OM	*cis*-linalool oxide	0.1	0.1
*16*	1086	MH	α-terpinolene	0.4	0.7
*17*	1097	OM	linalool	35.8	25.2
*18*	1158	OM	verbenol	0.1	0.1
*19*	1167	OM	terpinen-4-ol	0.4	0.5
*20*	1179	OM	α-terpineol	3.0	2.0
*21*	1198	OM	*cis*-geraniol	0.3	0.1
*22*	1245	OM	linalyl acetate	1.0	1.3
*23*	1255	OM	*trans*-geraniol	1.1	1.0
*24*	1296	OM	*trans*-pinocarvyl acetate	tr	tr
*25*	1318	OM	methyl geranate	tr	0.1
*26*	1347	OM	α-terpinyl acetate	0.7	0.9
*27*	1356	OM	nerol acetate	0.3	0.5
*28*	1376	OM	geranyl acetate	0.6	1.0
*29*	1402	OM	methyleugenol	0.5	1.0
*30*	1420	SH	β-caryophyllene	1.0	1.6
*31*	1436	SH	aromandendrene	tr	0.2
*32*	1457	SH	humulene	1.0	1.8
*33*	1517	OTHER	durohydroquinone	0.6	1.3
*34*	1588	OS	caryophyllene oxide	0.2	0.5
*35*	1603	OS	*trans*-bisabolene oxide	0.1	0.1
*36*	1617	OS	humulene oxide II	0.2	0.3
*37*	1660	OTHER	5,8,11-heptadecatrien-1-ol	tr	0.2
*38*	1705	OTHER	methyl ketostearate	0.1	0.4
			*Oil yield*	*0.96%*	*1.02%*
			**Total identified**	**100.0**	**99.2**
			**Monoterpene hydrocarbons (MH)**	**28.9**	**23.0**
			**Oxygenated monoterpenes (OM)**	**67.4**	**68.8**
			**Sesquiterpene hydrocarbons (SH)**	**2.0**	**3.5**
			**Oxygenated sesquiterpenes (OS)**	**0.4**	**0.9**
			**Aromatic hydrocarbons (AH)**	**0.3**	**0.1**
			**Other compounds (OTHER)**	**0.9**	**2.9**

The main common compounds are highlighted in grey color. LRI = Linear Retention Index, experimentally obtained on a HP-5MS column using a C_7_–C_30_ mixture of *n*-alkanes.

**Table 3:** GC-MS profiles of the essential oils obtained from the fruits of *Myrtus communis* subsp. *communis* collected in July (early fruiting) and in October 2019 (ripening stage).

	LRI	Class	Compounds	Relative Abundance (%)	
				*July*	*October*
*1*	770	OTHER	hexanal	0.2	-
*2*	841	OTHER	2-hexenal	0.4	-
*3*	911	OTHER	isobutyl isobutyrate	0.3	0.3
*4*	927	MH	α-thujene	3.3	2.0
*5*	936	MH	α-pinene	11.9	21.4
*6*	955	MH	camphene	0.2	-
*7*	980	MH	β-pinene	1.3	0.9
*8*	988	MH	myrcene	0.9	0.5
*9*	1007	MH	α-phellandrene	2.0	0.3
*10*	1010	MH	3-carene	6.5	3.8
*11*	1018	MH	α-terpinene	1.6	0.7
*12*	1029	AH	*o*-cymene	7.6	7.9
*13*	1035	MH	limonene	0.8	6.8
*14*	1042	OM	1,8-cineole	6.4	12.2
*15*	1049	MH	*E*-β-ocimene	4.3	1.3
*16*	1062	MH	γ-terpinene	5.1	4.0
*17*	1083	MH	isoterpinolene	0.1	Tr
*18*	1088	MH	α-terpinolene	5.2	3.9
*19*	1091	AH	*p*-cymenene	0.3	0.3
*20*	1101	OM	linalool	8.8	9.4
*21*	1111	OTHER	(*E*)-4,8-dimethylnona-1,3,7-triene	0.2	Tr
*22*	1125	OM	fenchol	0.1	Tr
*23*	1131	OM	*cis*-2-norbornanol	0.1	0.2
*24*	1149	OM	pinocarveol	0.1	0.1
*25*	1177	OM	isoborneol	0.2	0.3
*26*	1184	OM	terpinen-4-ol	1.9	0.8
*27*	1191	OM	ocimenol	0.3	-
*28*	1201	OM	α-terpineol	6.1	3.6
*29*	1218	OM	fenchyl acetate	tr	Tr
*30*	1229	OM	*cis*-geraniol	0.3	0.2
*31*	1247	OM	linalyl acetate	1.4	2.9
*32*	1252	OM	*trans*-geraniol	1.8	1.5
*33*	1286	OM	bornyl acetate	0.1	0.1
*34*	1320	OM	methyl geranate	0.3	0.2
*35*	1348	OM	α-terpinyl acetate	3.6	0.4
*36*	1356	OM	nerol acetate	0.4	0.3
*37*	1375	OM	geranyl acetate	0.9	0.7
*38*	1401	OM	methyleugenol	1.7	1.1
*39*	1424	SH	β-caryophyllene	4.3	2.6
*40*	1430	SH	γ-elemene	tr	-
*41*	1434	SH	aromandendrene	0.2	0.2
*42*	1461	SH	humulene	2.9	2.0
*43*	1494	SH	guaia-1(10),11-diene	0.2	0.2
*44*	1499	SH	bicyclogermacrene	0.2	0.1
*45*	1506	SH	3-carene, 4-acetyl	0.3	0.1
*46*	1518	OTHER	durohydroquinone	0.6	0.5
*47*	1521	SH	δ-cadinene	0.1	Tr
*48*	1566	SH	germacrene B	0.7	0.3
*49*	1574	OS	isoaromadendrene epoxide	tr	-
*50*	1586	OS	spathulenol	0.9	0.8
*51*	1589	OS	caryophyllene oxide	1.3	3.2
*52*	1594	OS	globulol	0.2	Tr
*53*	1607	OS	calarene epoxide	0.2	0.2
*54*	1619	OS	humulene epoxide II	0.6	1.5
*55*	1635	OS	aromandendrene epoxide	0.1	0.1
*56*	1644	OS	ledene oxide	0.3	0.3
			*Oil yield*	*0.59%*	*0.48%*
			**Total identified**	**99.5**	**100.0**
			**Monoterpene hydrocarbons (MH)**	**42.9**	**45.5**
			**Oxygenated monoterpenes (OM)**	**34.3**	**34.0**
			**Sesquiterpene hydrocarbons (SH)**	**8.9**	**5.5**
			**Oxygenated sesquiterpenes (OS)**	**3.7**	**6.0**
			**Aromatic hydrocarbons (AH)**	**7.9**	**8.2**
			**Other compounds (OTHER)**	**1.8**	**0.8**

The main common compounds are highlighted in grey color. LRI = Linear Retention Index, experimentally obtained on a HP-5MS column using a C_7_–C_30_ mixture of *n*-alkanes.

**Table 4:** Collection details of the analyzed plant samples of *Myrtus communis* subsp. *communis* in 2018 and 2019. March corresponds to the onset of the plant vegetative growth, July to the fruiting stage, October to the fruit ripeness.

Plant Material	*Fresh Leaves*	*−80 °C Frozen Leaves*	*−20 °C Frozen Leaves*	*Dried Leaves*	*Dried Unripe Fruits*	*Dried Ripe Fruits*
**Year 2018**	7 July	7 July	7 July	7 July	-	-
**Year 2019**	-	-	-	3 March	3 July	2 October
				2 October		

## Data Availability

Not applicable.

## Supplementary Materials

The following supporting information can be downloaded at: https://www.mdpi.com/article/10.3390/plants11060754/s1. Table S1. Chemical composition of the essential oils of *Myrtus communis* of different origin on the basis of literature data. From Hennia et al. [65], modified.

## Abbreviations

*DL* = Dried Leaves; EO = essential oil; *FL* = Fresh Leaves; *20FL* = Leaves frozen at −20 °C; *80FL* = Leaves frozen at −80 °C; GC-MS = Gas Chromatography-Mass Spectrometry; LRI = Linear Retention Index; MH = Monoterpene Hydrocarbons; OM = Oxygenated Monoterpenes; OS = Oxygenated Sesquiterpenes; NIST = National Institute of Standards and Technology; SH = Sesquiterpene Hydrocarbons.
