# Supplementary material for: Botanic Garden as a Factory of Molecules: Myrtus communis L. subsp. communis as a Case Study
Source: Plants (Basel). 2022 Mar 11;11(6):754. doi: 10.3390/plants11060754 (PMC8949965; doi:10.3390/plants11060754)
Supplement: Supplementary file 1 [file plants-11-00754-s001.zip › plants-1606265-supplementary.pdf]

Table S1. Chemical composition of the essential oils of *Myrtus communis* of different origin on the basis of literature data. From Hennia et al. [65], modified.

Plant material	Main compounds (relative abundances > 9.5%)	References
	**Albania**	
Leaves I:	α-Pinene (39.41), 1,8-cineole (32.31)	[66]
II:	α-Pinene (34.1), 1,8-cineole (36.57)	
III:	α-Pinene (43.74), 1,8-cineole (16.25), linalool (10.58)	
IV:	α-Pinene (45.54), 1,8-cineole (23.44)	
V:	α-Pinene (18.66), 1,8-cineole (13.80), (32.98)	
Leaves	α-Pinene (19.40–20.25), limonene (12.34–16.15), 1,8-cineole (16.63–21.77), linalool (8.79–13.37), myrtenyl acetate (11.35–12.26)	[8]
Flowers	α-Pinene (11.47–22.50), limonene (8.65–16.15), 1,8-cineole (13.79–15.15), linalool (9.64–16.70), myrtenyl acetate (16.85–17.66)	
Fruits	α-Pinene (17.23–21.46), limonene (12.63–15.07), 1,8-cineole (16.74–17.87), linalool (9.49–11.38), myrtenyl acetate (14.76–16.08)	
Leaves *Spring*	α-Pinene (18.33–30.99), 1,8-cineole (9.71–15.45), linalool (8.76–9.5)	[8]
Summer	α-Pinene (17.9–28.45), 1,8-cineole (7.42–10.75), linalool (8.76–10.93)	
Aerial parts	1,8-Cineole (31.19), α-pinene (22.95), linalool (12.14)	[67]
Leaves	α-Pinene (41.55), 1,8-cineole (32.24)	[68]
Leaves	1,8-Cineole (40.37), α-pinene (21.82)	[69]
Berries	1,8-Cineole (46.48), α-pinene (24.52)	
Leaves Chemotype A:	1,8-cineole (52.5), myrtenyl acetate (26.3), α-pinene (9.5)	[70]
Chemotype B:	1,8-cineole (52.2), α-pinene (29.7)	
Chemotype C:	1,8 cineole (32.0), methyl eugenol (33.6)	
Chemotype D:	myrtenyl acetate (61.1)	
Chemotype E:	1,8-cineole (37.4), α-terpinolene (20.8), α-terpineol (16.5)	
Aerial parts	Borneol (27.15), 1,8-cineole (21.33), α-pinene (11.09), camphene (9.49)	[71]
	**Croatia**	
Leaves	α-Pinene (6.6–16.4), 1,8-cineole + limonene (12.6–29.8), linalool (10.8–18.3), myrtenyl acetate (13.5–30.7)	[72]
Flowers	1,8-cineole + limonene (8.1–11.9), myrtenyl acetate (23.6–29.3)	
Fruits	α-Pinene (4.0–14.4), 1,8-cineole + limonene (10.9–21.1), myrtenyl acetate (12.2–33.2)	
	**Montenegro**	
Field-grown plants	α-Pinene (21.3), 1,8-cineole (22.0), linalool (12.0), myrtenyl acetate (13.4)	[73]
Leaves I	1,8-Cineole (13.280), linalool (22.276), myrtenyl acetate (16.561)	[74]
Leaves II	1,8-Cineole (16.878), linalool (18.320), myrtenyl acetate (18.009)	
Leaves III	1,8-Cineole (15.762), linalool (26.591), myrtenyl acetate (18.489)	
	**Italy**	

Leaves I	α-Pinene (19.9), 1,8-cineole (57.2)	[15]
Fruits I	α-Pinene (47.2), 1,8-cineole (14.6)	
Leaves II	α-Pinene (9.5), 1,8-cineole (33.6), β-caryophyllene (11.7)	
Fruits II	α-Pinene (24.7), β-elemene (17.2), β-caryophyllene (14.4)	
Leaves and flowers	α-Pinene (15.7), 1,8-cineole (16.5), linalyl acetate (13.2)	[16]
Leaves	α-Pinene (30.0-59.5), limonene (5.2-29.8), 1,8-cineole (15.9-41.7)	[11]
Berries	α-Pinene (18.2-38.9), limonene (5.0-44.5), 1,8-cineole (5.8-24.8)	
Leaves	α-Pinene (9.5-26.8), linalool (9.8-28.9), 1,8-cineole (10.9-26.6), α-terpineol (3.8-22.9)	[12]
Flowers	α-Pinene (9.9-11.1), linalool (6.8-15.9), 1,8-cineole (4.5-29.1), α-terpineol (7.7-18.3)	
Leaves	α-Pinene (10.5-57.9), linalool (3.7-38.4), 1,8-cineole (7.7-33.3)	[18]
Leaves	α-Pinene (9.4-48.5), linalool (2.3-29.6), 1,8-cineole (4.5-18.9)	[17]
	**Portugal**	
Leaves	α-Pinene (43.5–43.7), limonene (15.0–15.5), 1,8-cineole (22.9–25.3)	[75]
Leaves	α-Pinene (10.38–21.5), limonene+1,8-cineole (20.03–39.45), myrtenyl acetate (7.40–37.62)	[76]
Aerial parts	1,8-Cineole (36.7), α-pinene (17.3), myrtenyl acetate (17.1), limonene (9.8)	[77]
	**Chipre**	
Leaves	1,8-Cineole (50.12), linalool (12.65)	[78]
	**Turkey**	
Leaves	1,8-Cineole (18.2), linalool (16.3), myrtenyl acetate (14.5)	[79]
Leaves and branches	1,8-Cineole (10.5), linalool (18.6), myrtenyl acetate (10.8)	
Leaves and flowers	1,8-Cineole (37.03), α-pinene (30.17)	[80]
Leaves	Linalool (31.3), linalyl acetate (17.8), 1,8-cineole (14.7) (57,68)	
Leaves	1,8-Cineole (37), α-pinene (30.2), linalool (9.7)	[81]
Leaves	α-Pinene (29.4), limonene (23.5), 1,8-cineole (20.1), linalool (10.4)	[82]
Fruits	1,8-Cineole (29.20), linalool (15.67), α-terpineol (18.43)	[83]
Leaves	1,8-Cineole (49.15), myrtenol (19.49)	[84]
	**Egypt**	
Leaves	1,8-Cineole (27.2), α-pinene (25.5), linalool (11.8)	[85]
Fruits	1,8-Cineole (29.6), menthyl acetate (13.4), *trans*-caryophyllene (10.5)	
Leaves	α-Pinene (26.16), 1,8-cineole (16.45), linalool (11.23)	[86]
Leaves	α-Pinene (26.16), 1,8-cineole (16.45), linalool (11.23)	[87]
Leaves	α-Pinene (18.0), limonene (21.8), linalyl acetate (31.4)	[88]
Leaves	α-Pinene (10.1–11.6), 1,8-cineole (12.7–19.6), linalool (7.0–15.8), myrtenyl acetate (23.7–39.0)	[89]

Leaves of cultivated plants during the fruiting stage	limonene (14.5), linalool (15.6), myrtenyl acetate (24.4)	[90]
Leaves of the wild plants, during the flowering stage	Myrtenyl acetate (31.8), α-pinene (29.8), 1,8-cineole (10.3)	[91]
Aerial parts	α-pinene (16.0), 1,8-cineole (11.4), linalool (13.7), myrtenyl acetate (19.7)	[90]
	**Algeria**	
Leaves	1,8-Cineole (46.98), cis-geraniol (25.18)	[92]
Leaves	Hydrodistillation: α-Pinene (44.62), 1,8-cineole (25.46) (33)<br>Solvent-free-microwave-extraction: α-Pinene (30.65), 1,8-cineole (32.12)	[9]
Leaves	α-Pinene (46.9), 1,8-cineole (25.2)	[93]
Berries	1,8-Cineole (11.4), linalool (36.2), estragole (18.4)	
Leaves	α-Pinene (32.8–48.4), limonene (6.0–23.9), 1,8-cineole (6.5–30.4)	[9]
Leaves	α-Pinene (39.3), 1,8-cineole (33.3)	[94]
Twigs	α-Pinene (10.8), 1,8-cineole (13.5)	
Leaves	1,8-Cineole (26.2), α-Pinene (18.96), limonene (11.12)	[95]
Leaves and flowers	Collective sample I:α-Pinene (50.8), 1,8-cineole (21.9)	[96]
	Collective sample II:α-Pinene (33.6), 1,8-cineole (13.3), linalool (14.8), linalyl acetate (9.5)	
Leaves	Limonene (23.4), linalool (15.4), α-pinene (10.7), geranyl acetate (10.9)	[97]
Berries	Limonene (12.93), octadienol (12.85), α-pinene (10.01)	[98]
Leaves	α-Pinene (22.8–38.7), 1,8-cineole (10.3–24.2), limonene (11.0–18.7), linalool (1.8–32.0)	[99]
Fruits	α-Pinene (21.1–43.4), 1,8-cineole (5.2–31.3), limonene (11.0–18.7)	
	**Tunisia**	
Leaves	1,8-Cineole (61.0), α-pinene (23.7)	[100]
Leaves	α-Pinene (45.9–52.29), 1,8-cineole (19.0–23.1), limonene (9.0–9.7)	[101]
Leaves	α-Pinene (8.63–26-73), 1,8-cineole (25.62), linalool (3.91–11.18), linalyl acetate (0.99–12.96), α-terpineol (1.96–28.44)	[102]
Fruits	α-Pinene (1.24–12.64), 1,8-cineole (7.31–40.99), geranyl acetate (1.83–20.54), β-caryophyllene (0.85–10.83)	[103]
Leaves	α-Pinene (58.05), 1,8-cineole (21.67)	[23]
Flowers	α-Pinene (17.53), 1,8-cineole (12.70), limonene (10.11), eugenol (10.11)	
Stems	1,8-Cineole (32.84), α-pinene (10.53), (E)-β-ocimene (9.48)	
Leaves	α-Pinene (16.72–30.70), 1,8-cineole (20.12–30.64), linalool (10.44–11.80), myrtenyl acetate (0.14–20.18)	[104]
Leaves	α-Pinene (21.6–39.4), limonene (0.1–18.8), 1,8-cineole (12.5–26.3), linalool (4.6–20.0)	[105]
Fruits	1,8-Cineole (23.7), linalool (15.3)	

Floral buds	α-Pinene (48.9), 1,8-cineole (15.3)	[23]
Leaves	α-Pinene (52.2), 1,8-cineole (21.9)	[106]
Fruits	α-Pinene (1.73–10.36), 1,8-cineole (13.27–25.95), linalool (12.79–15.55), α-terpineol (5.42–12.51), geranyl acetate (5.08–10.21)	[107]
Leaves	α-Pinene (15.59), limonene (8.94), 1,8-cineole (16.55), linalool (13.30), myrtenyl acetate (20.75)	[108]
Leaves	α-Pinene (35.60), 1,8-cineole (29.60)	[109]
Leaves	α-Pinene (55.66), 1,8-cineole (30.05)	[110]
Leaves	α-Pinene (44.1), 1,8-cineole (36.0)	[111]
	**Morocco**	
Not reported	1,8-Cineole (74.5), α-pinene (9.5)	[112]
Leaves	1,8-Cineole (43.03), myrtenyl acetate (25.05), α-pinene (10.00)	[113]
Leaves and flowers	1,8-Cineole + limonene (40.9), α-pinene (21.8), myrtenyl acetate (19.8)	[114]
Leaves	Myrtenyl acetate (49.27), 1,8-cineole (26.93), α-pinene (16.52)	[115]
Aerial parts	α-Pinene (10.0), 1,8-cineole (43.1), myrtenyl acetate (25.0)	[116]
Leaves	α-Pinene (5.7–25.7), limonene (9.5–11.4), 1,8-cineole (29.7–32.4), myrtenyl acetate (14.9–33.0)	[76]
	**Iran**	
Not reported	α-Pinene (29.1), limonene (21.5), 1,8-cineole (17.9), linalool (10.4)	[117]
Aerial parts	Limonene (38.2), α-pinene (31.5)	[118]
Not reported	α-Pinene (29.4), limonene (21.2), 1,8-cineole (18.0)	[119]
Leaves	1,8-Cineole (36.1), α-pinene (22.5), linalool (8.4)	[30]
Not reported	α-Pinene (29.4), limonene (21.2), 1,8-cineole (18.0), linalool (10.66)	[120]
Leaves at the flowering stage	α-Pinene (3.8–23.0), 1,8-cineole (9.9–20.3), limonene (5.5–17.8), linalool (12.3–17.6)	[121]
Ripe fruits	1,8-Cineole (24.0), α-pinene (22.1), limonene (17.6), linalool (11.4) (155)	
Leaves	α-Pinene (29.9–38.6), limonene (13.5–18.1), 1,8-cineole (23.3–29.1)	[122]
Leaves	α-Pinene, 1,8-cineole, myrtenyl acetate	[30]
Leaves and flowers	α-Pinene (47.8), 1,8-cineole (25.9)	[124]
Leaves	α-Pinene (38.71–56.3), limonene (12.01–38.0), 1,8-cineole (0–18.92), linalool (2.82–11.5)	[125]
Leaves	α-Pinene (16.62–47.83), limonene (0–24.4), 1,8-cineole (9.56–28.94), linalool (9.17–15.76)	[126]
Leaves	α-Pinene (39.2), 1,8-cineole (22.0), linalool (18.4)	[127]
Leaves	α-Pinene (24.42–31.57), limonene (tr-23.41), 1,8-cineole (5.92–21.21), linalool (8.72–11.56)	[128]
Leaves	α-Pinene (22.3–38.8), limonene (tr-21.4), 1,8-cineole (8.7–43.8), linalool (6.4–14.5)	[129]
Leaves and flowers	α-Pinene (23.0), 1,8-cineole (20.3), limonene (17.8), linalool (12.3)	[130]
Leaves	α-Pinene (24.4–30.8), limonene (31.4–44.8), 1,8-cineole (11.2–22.3)	[131]
Leaves	α-Pinene (17.5–37.1), 1,8-cineole (9.9–29.8), linalool (7.0–23.1), linalyl acetate (2.3–10.5)	[132]

Leaves	α-Pinene (7.04–31.29), limonene (14.02–22.52), linalool (1.72–15.47)	[133]
Leaves or aerial parts	α-Pinene (27.24–52.39), 1,8-cineole (24.79–37.99)	[134]
Leaves	α-Pinene (27.87), 1,8-cineole (20.15), linalool (10.26)	
Leaves	α-Pinene (22.02), 1,8-cineole (26.91), linalool (12.74)	[135]
Shoots	α-Pinene (32.5), 1,8-cineole (24.15), limonene (15.5), linalool (10.6)	[136]
	**Saudi Arabia**	
Aerial parts	α-Pinene (11.6), 1,8-cineole (26.5), linalool (18.0)	[137]
	**Yemen**	
Leaves	Linalool (29.1), 1,8-cineole (18.4), α-terpineol (10.8)	[138]
